# The Pharmacological Assessment of GABA_A_ Receptor Activation in Experimental Febrile Seizures in Mice

**DOI:** 10.1523/ENEURO.0429-18.2019

**Published:** 2019-03-04

**Authors:** Yuka Kasahara, Hideyoshi Igata, Takuya Sasaki, Yuji Ikegaya, Ryuta Koyama

**Affiliations:** 1 Laboratory of Chemical Pharmacology, Graduate School of Pharmaceutical Sciences, University of Tokyo, Tokyo 113-0033, Japan; 2 Department of Molecular Pharmacology and Neurobiology, Yokohama City University Graduate School of Medicine, Yokohama 236-0004, Japan; 3 Precursory Research for Embryonic Science and Technology, Japan Science and Technology Agency, Kawaguchi, Saitama 332-0012, Japan; 4 Center for Information and Neural Networks, Suita City, Osaka 565-0871, Japan

**Keywords:** excitatory GABA, febrile seizure, GABA, LFP

## Abstract

Hyperthermia-induced febrile seizures (FSs) are the most common seizures during childhood, and prolonged complex FSs can result in the development of epilepsy. Currently, GABA_A_ receptor modulators such as benzodiazepines and barbiturates are used as medications for FSs with the aim of enhancing GABA-mediated inhibition of neuronal activity. However, it is still up for debate whether these enhancers of GABAergic neurotransmission could depolarize immature neurons with relatively higher levels of the intracellular Cl^−^ in the developing brain during FSs. Here, we performed simultaneous video-local field potential monitoring to determine whether benzodiazepines and barbiturates affect the phenotypes of FSs in postnatal day (P)11 and P14 mice. We found that low-dose administration of diazepam decreased the incidence of clonic seizures at P11. We also found that high-dose administration of diazepam and pentobarbital exacerbated the behavioral and electrophysiological phenotypes of the induction phase of experimental FSs at P11 but not at P14. We further found that the deteriorated phenotypes at P11 were suppressed when Na^+^K^+^2Cl^−^ cotransporter isoform 1 (NKCC1), which mediates Cl^−^ influx, was blocked by treatment with the diuretic bumetanide. Though our findings do not exclude the involvement of sedation effect of high-dose GABA_A_ receptor modulators in worsening experimental FSs at P11, pharmacological enhancement of GABAergic signaling could aggravate seizure activity in the early phase of FSs.

## Significance Statement

Febrile seizures are the most common neurologic disorder in children. For the treatment of febrile seizures, GABA_A_ receptor modulators are generally used and successfully suppress seizures in most cases. However, it is still up for debate whether these drugs could depolarize immature neurons in the developing brain. Here, using a mouse model of febrile seizures, we show that high-dose GABA_A_ receptor modulators exacerbate the behavioral and electrophysiological phenotypes of complex febrile seizures at postnatal day (P)11 but not at P14. We further found that the Na^+^K^+^2Cl^−^ cotransporter isoform 1 blocker bumetanide suppresses the phenotypes deteriorated by GABA_A_ receptor modulators. Thus, our findings suggest high-dose GABA_A_ receptor modulators possibly activate immature neurons.

## Introduction

Hyperthermia-induced febrile seizures (FSs) are the most common convulsive event in infancy and childhood (Koyama and Matsuki, 2010, Koyama et al., 2012). Approximately 60–70% of FSs are considered to be benign, but the remaining 30–40% of FSs with prolonged duration (>15 min), recurrent seizures or focal neurologic features are classified as complex. FSs are associated with the development of temporal lobe epilepsy (Cendes et al., 1993; French et al., 1993). Thus, proper medication for complex FSs, especially when the seizures progress to febrile status epilepticus, is necessary (Berg and Shinnar, 1996; Lewis et al., 2014; Seinfeld et al., 2014).

FSs are usually treated with benzodiazepines and occasionally with barbiturates (Khosroshahi et al., 2011; Camfield and Camfield, 2014; Salehiomran et al., 2016). The GABA_A_ receptor complex is a pentameric heterooligomer that contains binding sites for benzodiazepines and barbiturates. Benzodiazepines, including diazepam, enhance the binding of GABA to GABA_A_ receptors, increasing the frequency of chloride channel opening; barbiturates, including pentobarbital, prolong the open time of the chloride channel directly (Study and Barker, 1981; Treiman, 2001). These GABA_A_ receptor modulators are used as treatment for FSs in an attempt to enhance GABA-mediated inhibition of neuronal activity. In most cases, diazepam has been shown to be effective to suppress seizures in children with complex FSs (Appleton et al., 1995; Chamberlain et al., 1997; Lahat et al., 2000; Mahmoudian and Zadeh, 2004). However, one major question still remains is that GABA_A_ receptor modulators potentially activate neurons during early-life seizures because GABA can be depolarizing, i.e., excitatory, in the developing brain, where immature neurons overwhelm mature neurons. Using experimental approaches in the developing rodent brain, it has been reported that GABAergic signaling depolarizes neurons and contributes to the initiation of ictal epileptiform activity and the generation of spontaneous seizures (Dzhala and Staley, 2003; Khalilov et al., 2003, 2005). The effects of pharmacological activation of GABA_A_ receptors during experimental FSs have not been fully evaluated because it is difficult to perform stable electroencephalographic recordings in freely moving (seizing) postnatal mice with small brains and fragile skulls.

Whether GABA_A_ receptor activation depolarizes or hyperpolarizes neurons depends on intracellular Cl^−^ levels and the Cl^−^ equilibrium potential, which are mainly controlled by cation-chloride cotransporters (CCCs); one such CCC is the Na^+^-K^+^-Cl^−^ cotransporter (NKCC) 1, which mediates Cl^−^ influx (Fukuda et al., 1998; Ben-Ari, 2002; Payne et al., 2003). Thus, early expression of NKCC1 partly contributes to the excitatory action of GABA in immature neurons because of an elevated intracellular Cl^−^ level and a depolarized Cl^−^ equilibrium potential. Bumetanide, the selective inhibitor of NKCC1, which has been shown to be potentially useful for the treatment of epilepsy (Löscher et al., 2013a, b), decreased seizure events and susceptibility after early-life seizures in some animal models, including FS models, either alone or with phenobarbital (Dzhala et al., 2005, 2008, 2010; Nardou et al., 2011; Koyama et al., 2012; Cleary et al., 2013; Holmes et al., 2015; Hu et al., 2017). However, it remains unclear how the pharmacological activation of GABA_A_ receptors and the inhibition of the NKCC1 transporter affects behavioral phenotypes and neuronal activity during FSs.

In the present study, we developed a system to stably perform simultaneous video-local field potential (LFP) monitoring in postnatal mice to evaluate whether benzodiazepines and barbiturates affect the phenotypes of FSs. Using this system, we have also examined the effects of bumetanide with or without GABA_A_ receptor modulators on FS phenotypes.

## Materials and Methods

### Animal ethics

Male and female C57BL/6J mice were purchased from SLC and treated under the approval of the animal experiment ethics committee at the University of Tokyo (approval number: P29–14) and the guidelines for the care and use of laboratory animals. All efforts were made to minimize the animals’ suffering and the number of animals used.

### Mouse model of complex FSs

Complex FSs were induced by exposing postnatal day (P)11 mice to hyperthermia (Bender et al., 2004; Koyama et al., 2012, Koyama, 2017; Tao et al., 2016; Kasahara et al., 2018). Hyperthermic conditions were maintained via a regulated stream of moderately heated air. The core temperature of the mice was raised gradually and measured as rectal temperature every 2 min. A rectal temperature between 39.5 and 43°C was maintained for 30 min. The definition of clonus seizures was behavioral seizures with hindlimb clonus with falling. Drugs were administered 15 min before the induction of hyperthermia.

### Animal surgery for electrode implantation

P10 mice were anaesthetized with isoflurane gas (0.5–1.5%); lidocaine (0.043 mg/kg) was given subcutaneously as an analgesic. Electrodes for LFP recordings were implanted into the medial parietal association cortex or the primary somatosensory trunk at 1.9 mm posterior and 0.9 mm lateral to bregma at a depth of 1.0 mm. A ground/reference electrode was placed into the frontal cortex. After the surgery, mice recovered from the anesthesia and were placed on a heat plate overnight to maintain body temperature.

### Simultaneous electrophysiological recording and video monitoring

Electrophysiological recordings were performed using a weight-saving modified electrophysiological recording system (8200 system Series, Pinnacle Technology) and PAL 8200 software (Pinnacle Technology). Data were collected at a sampling rate of 1000 Hz and low-pass filtered at a cutoff frequency of 500 Hz. LFP signals were digitalized within a range from −4.0 to +4.0 mV. When recording, the mouse implanted with electrodes was placed in a cylinder-shaped glass container (11 cm in diameter, 17 cm in height), and a recording was started after a 10 min habituation period. After recording data in a 30 min pre-hyperthermia period, the mouse was subjected to the hyperthermia protocol. Recording lasted for up to 50 min after the induction of hyperthermia. Of 29 mice tested, 17 mice died during the 50 min recording period. Rectal temperature was measured every 2 min.

### Histologic analysis to confirm electrode locations

After the electrophysiological recordings, the mice were perfused with cold PBS followed by 4% paraformaldehyde (PFA). Brain samples were postfixed with 4% PFA overnight and rinsed three times with 0.1 m PBS. The brain samples were sectioned into 100-µm-thick horizontal slices using a DTK-1500 vibratome. The samples were subsequently incubated with Hoechst in PBS at room temperature for 10 min with agitation to reveal neuronal cytoarchitecture. Samples were rinsed three times with 0.1 m PBS and embedded in Permafluor (ThermoFisher Scientific). Representative images were acquired using a BZ-X700 microscope and analyzed using ImageJ (NIH). *Z*-series images were collected with a 0.1 NA 2× objective at a voxel size of 7.6-7.6-55 µm (*x*-*y*-*z*) for the representative images.

### Detection of large amplitude events in LFP signals

All of the LFP analyses were conducted using Python. LFP data collected during the periods of measuring rectal temperature were excluded from all analyses. To reduce humming noise, a 49–51 Hz notch filter was applied to the LFP data. The envelope of filtered LFP traces was computed via Hilbert transformation and then Gaussian-filtered with a 5 ms kernel. In each mouse, LFP signals at a pre-hyperthermia baseline were quantified by calculating an average (mean) and a SD from a dataset including the bottom 25% of the LFP envelopes obtained before inducing hyperthermia. The mean and SD were 40.7 ± 2.0 and 5.3 ± 0.3 μV, respectively (*n* = 29 mice). In a series of LFP envelopes within an animal, all of the large deflections of the LFP signals that possibly contained both seizure-induced neuronal discharges and simple movement artifacts were first extracted and then termed large amplitude (LA) events. An LA event was detected when an LFP envelope exceeded a threshold of the mean + 50×SD after the induction of hyperthermia. The onset and offset of the LA event were marked at the points when the LFP envelopes first exceeded the mean + 20×SD and then dropped below the mean + 20×SD, respectively. LA events with a duration of <10 ms were excluded. If an inter-event interval between two LA events was <200 ms, the neighboring two events were detected as a single LA event. Next, LA events were further scrutinized by the following three criteria: (1) The absolute values of the LFP traces below the mean + 50×SD were converted to 0, and the remaining above-threshold absolute LFP traces were Gaussian-filtered with a 5 ms kernel. For each LA event, the first peak was detected from the filtered trace. LA events in which the first peaks of the filtered trace were below the mean + 50×SD and in which subsequent peaks were detected within 20 ms after the first peak were excluded from further analyses. (2) For each LA event, a rise time was calculated as the period between the initial point that crossed 0 before the first peak in the original LFP trace and the first peak. Events with a rise time of <10 and >200 ms were excluded from further analyses. These two criteria specifically removed events with high-frequency zigzag-shaped traces. Of all the LA events, 81.2% (4816/5931) and 80.1% (2879/3595) of the events before and during hyperthermia met these criteria; they had an average rise time of 31.9 ± 0.3 and 27.5 ± 0.3 ms before and during hyperthermia, respectively. (3) For each LA event, a sharpness index that represented how sharply the signal reached the first peak was computed as the ratio of the number of positive differentiated values to the number of negative differentiated values within a rise time in the absolute LFP trace. Events with a sharpness index of <2 were excluded from further analyses. In the end, 45.5% (2193/4816) and 62.3% (1793/2879) of the events before and during hyperthermia met the third criterion; they had an average rise time of 21.8 ± 0.2 and 22.5 ± 0.3 ms before and during hyperthermia, respectively. Among the LA events that met all of the above criteria, events in which the amplitude of the first peak was >3 mV were specifically extracted as epileptic events unless otherwise specified. The duration of an epileptic event was defined as the period between its onset and offset. This analysis was modeled on a previous human electroencephalogram study (Kane et al., 2017) that analyzed epileptiform discharges, spikes (20–70 ms), or sharp waves (70–200 ms).

### Statistical analysis

The data are presented as the mean ± SEM. Data collection and statistical tests were performed by researchers blinded to the experimental conditions. Data-labels were randomized before the analysis. The significance of the observed differences among saline and drug treatment groups was evaluated by Tukey’s test after one-factor ANOVA.

## Results

### GABA_A_ receptor modulators increase the severity of FSs in mice

**Figure 1.**
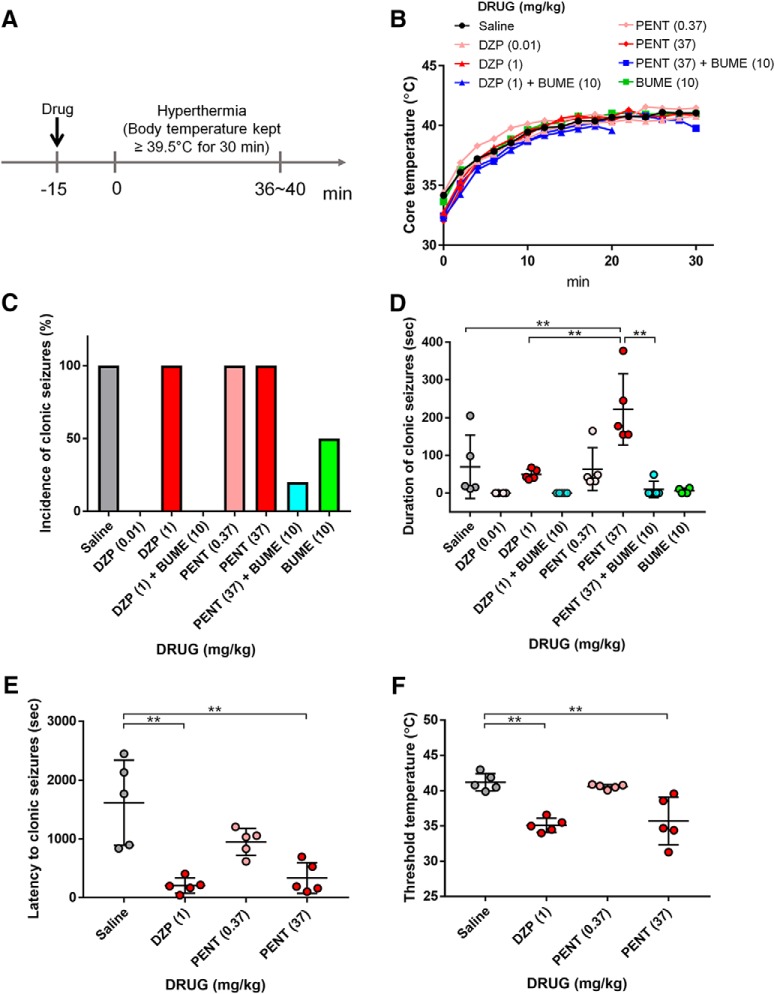
GABA_A_ receptor modulators increase susceptibility to FSs in P11 mouse. ***A***, Experimental timeline of the induction of prolonged FSs. ***B***, The elevation pattern of temperature during hyperthermia with the treatment of saline, pentobarbital, diazepam, and bumetanide; *n* = 4–5 mice. ***C***, The incidence of clonus seizures per mouse with the treatment of saline, pentobarbital, diazepam, and bumetanide; *n* = 4–5 mice (saline, 5/5; 0.01 mg/kg diazepam, 0/4; 1 mg/kg diazepam, 5/5; 1 mg/kg diazepam + 10 mg/kg bumetanide, 0/5; 0.37 mg/kg pentobarbital, 5/5; 37 mg/kg pentobarbital, 5/5; 37 mg/kg pentobarbital + 10 mg/kg bumetanide, 1/5; 10 mg/kg bumetanide, 2/4). ***D***, Normalized duration of clonus seizures induced by hyperthermia. Data represent the mean ± SEM. *p < 0.05, **p < 0.01, Tukey’s test; *n* = 4–5 mice. ***E***, Latency of clonus seizures induced by hyperthermia. Data represent the mean ± SEM. *p < 0.05, **p < 0.01 versus saline, Tukey’s test; *n* = 5 mice. ***F***, Threshold temperature of clonus seizures induced by hyperthermia. Data represent the mean ± SEM. **p < 0.01 versus saline, Tukey’s test; *n* = 5 mice.

**Figure 2.**
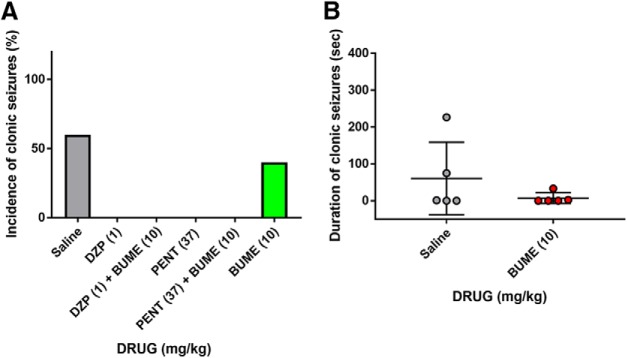
GABA_A_ receptor modulators decrease susceptibility to FSs in P14 mouse. ***A***, The incidence of clonus seizures per mouse with the treatment of saline, pentobarbital, diazepam, and bumetanide; *n* = 5 mice (saline, 3/5; 1 mg/kg diazepam, 0/5; 1 mg/kg diazepam + 10 mg/kg bumetanide, 0/5; 37 mg/kg pentobarbital, 0/5; 37 mg/kg pentobarbital + 10 mg/kg bumetanide, 0/5; 10 mg/kg bumetanide, 2/5). ***B***, Normalized duration of clonus seizures induced by hyperthermia. Data represent the mean ± SEM. Tukey’s test; *n* = 5 mice.

To determine the effects of GABA_A_ receptor modulators and the NKCC1 inhibitor on the behavioral phenotypes of hyperthermia-induced FSs, we treated mice at P11 with diazepam, pentobarbital, and bumetanide 15 min before FS induction (Fig. 1*A*). Drugs were injected subcutaneously, and their concentration was determined according to previous studies using rodents: pentobarbital (0.37 or 37 mg/kg; Dubé et al., 2006); diazepam (0.01 or 1 mg/kg; Liljelund et al., 2005); and bumetanide (10 mg/kg; Töllner et al., 2014). FSs were induced by raising the core temperature of the mice to between 39.5 and 43°C in a warm air stream-induced hyperthermic environment (Fig. 1*A*,*B*; Koyama, 2017). The number of mice that exhibited clonic seizures, a typical seizure phenotype in rodent FS models, during 30 min of the hyperthermic condition was comparable between the saline controls and the GABA_A_ receptor modulator group; the incidence of clonic seizures decreased when 10 mg/kg bumetanide was coadministered (Fig. 1*C*; Table 1). In addition, we found that the duration of the clonic seizures was significantly increased compared with controls when 37 mg/kg pentobarbital was administered; this increase was blocked when bumetanide was coadministered (Fig. 1*D*). These results suggest that high doses of GABA_A_ receptor modulators could exacerbate FS phenotypes, likely by enhancing depolarizing GABA_A_ receptor signaling. However, it should be noted that the incidence of clonic seizures decreased in P14 mice (Fig. 2; Table 2) and that the administration of high-dose diazepam or pentobarbital decreased the incidence of clonic seizures (Fig. 2; Table 2). These results suggest that the GABA excitatory/inhibitory shift presumably takes place at ∼P11–P14 in mice.

**Table 1.** Basic characteristics of P11 mice in individual drug-treated groups used for hyperthermia tests

	**Saline**	**PENT**			**DZP**			**BUME**
Conc, mg/kg	—	0.37	37	37	0.01	1	1	10
BUME, +/−	−	−	−	+	−	−	+	+
Body weight at P11, g	5.4 ± 0.26	4.5 ± 0.12	5.3 ± 0.16	5.3 ± 0.12	6.0 ± 0.17	5.6 ± 0.15	5.0 ± 0.19	5.2 ± 0.20
Seizure	5/5	5/5	5/5	1/5	0/4	5/5	0/5	2/4
Death	0/5	0/5	0/5	1/5	0/4	5/5	5/5	1/4
Average temp, °C	39.6 ± 0.46	40.0 ± 0.47	39.4 ± 0.57	37.7 ± 0.02	39.4 ± 0.40	38.8 ± 0.73	37.7 ± 0.02	39.6 ± 0.50
Highest temp, °C	42.1 ± 0.30	42.2 ± 0.16	42.1 ± 0.07	41.1 ± 0.16	41.2 ± 0.16	41.3 ± 0.18	40.2 ± 0.17	41.6 ± 0.14

The first three rows represent the types of drugs used (PENT, pentobarbital; DZP, diazepam; BUME, bumetanide), the concentration (Conc) of the drugs, and whether 10 mg/kg bumetanide was coadministered. The bottom rows show the average body weight at P11, the number of animals exhibiting seizures out of the total number of animals tested, the average rectal temperature during hyperthermia induction, and the highest rectal temperature during hyperthermia.

**Table 2.** Basic characteristics of P14 mice in individual drug-treated groups used for hyperthermia tests

	**Saline**	**PENT**		**DZP**		**BUME**
Conc, mg/kg	—	37	37	1	1	10
BUME, +/−	−	−	+	−	+	+
Body weight at P14, g	6.1 ± 0.13	6.4 ± 0.095	6.3 ± 0.13	6.4 ± 0.073	6.2 ± 0.17	6.3 ± 0.10
Seizure	3/5	0/5	0/5	0/5	0/5	2/5
Mortality	0/5	0/5	0/5	5/5	5/5	5/5
Average temp, °C	40.7 ± 0.45	39.8 ± 0.64	39.7 ± 0.63	40.3 ± 0.56	39.6 ± 0.68	40.7 ± 0.65
Highest temp, °C	42.8 ± 0.27	42.0 ± 0.18	42.1 ± 0.15	42.3 ± 0.13	42.3 ± 0.26	42.6 ± 0.055

The first three rows represent the types of drugs used (PENT, pentobarbital; DZP, diazepam; BUME, bumetanide), the concentration (Conc) of the drugs, and whether 10 mg/kg bumetanide was coadministered. The bottom rows show the average body weight at P14, the number of animals exhibiting seizures out of the total number of animals tested, the average rectal temperature during hyperthermia induction, and the highest rectal temperature during hyperthermia.

We also examined the threshold temperature and latency to clonus seizures in the four groups in which the incidence of clonus seizures was 100% (Fig. 1*E*,*F*) because these parameters reflect susceptibility to FSs. We found that 1 mg/kg diazepam and 37 mg/kg pentobarbital decreased the latency to clonic seizures (Fig. 1*E*). Furthermore, the GABA_A_ modulators were prone to decrease the threshold temperature to clonic seizures (Fig. 1*F*). These results suggest that enhanced GABA_A_ receptor signaling increases seizure susceptibility to hyperthermia. It should be noted that some studies reported that pentobarbital prevented hyperthermia-induced seizures in rat FS models (Dube et al., 2000; Brewster et al., 2002; Koyama et al., 2012). In these reports, pentobarbital was injected before the induction of hyperthermia as a hyperthermic control to distinguish whether observed phenomena in FS models was induced by seizures or by hyperthermia itself. It is possible that the difference in animal species (mice vs rats) results in the difference in sensitivity to pentobarbital.

### Development of a local field recording system in the postnatal mouse brain

**Figure 3.**
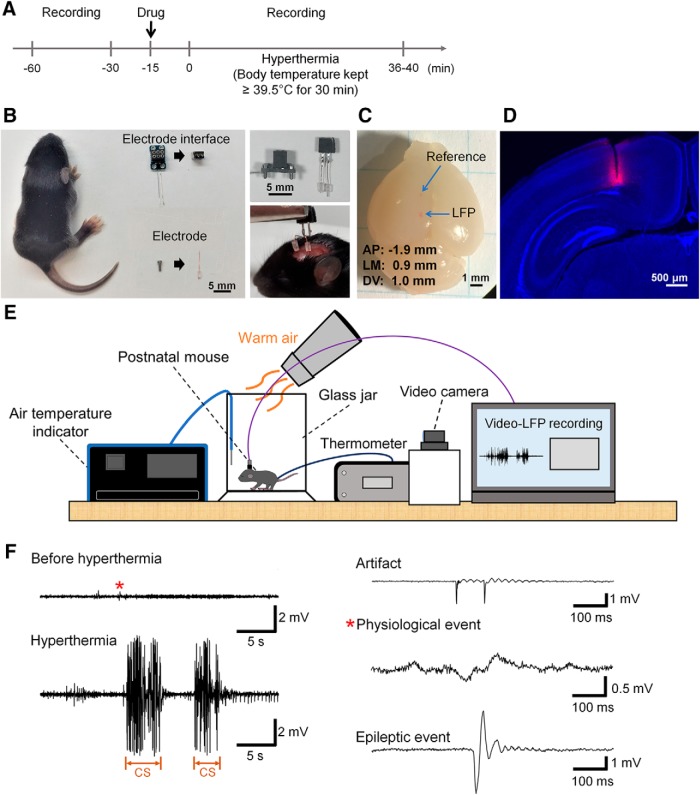
Cortical LFP recordings from a neonatal mouse subjected to hyperthermia induction. ***A***, Experimental timeline of LFP recordings. The drug was administered 15 min before the induction of hyperthermia. ***B***, Images of electrodes and electrode interfaces used for LFP recordings and a P10 mouse. The recording devices on the left were ready-made and those on the right were improved. ***C***, Top view of the mouse brain showing the positions of an LFP recording electrode (bottom, medial parietal association cortex or primary somatosensory trunk) and a reference electrode (top, frontal cortex). AP, Anteroposterior; LM, lateromedial; DV, dorsoventral. ***D***, Histologic verification of a recording site labeled with DiI in a coronal brain section. ***E***, A schematic image of the recording system. ***F***, Left, Representative LFP trace before hyperthermia with typical physiologic events (indicated by asterisk) and a representative LFP trace during hyperthermia with epileptiform discharges time-locked to the occurrence of chronic seizures (CSs) are shown. Right, Representative LFP traces of artifact, physiologic event and epileptic event are shown.

**Figure 4.**
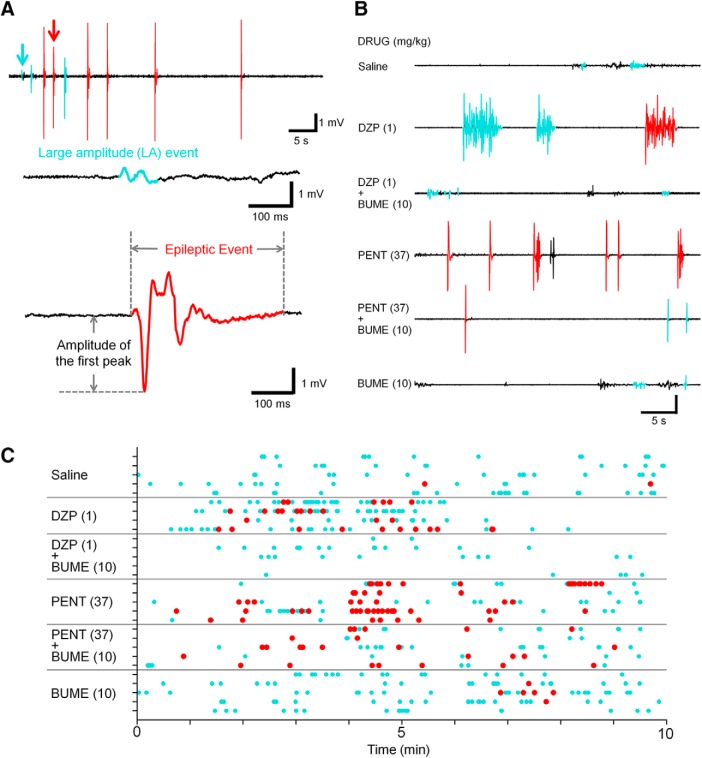
GABA_A_ receptor modulators increase epileptic events 10 min after the induction of hyperthermia. ***A***, Top, Representative LFP trace in the early period of hyperthermia under the administration of pentobarbital (37 mg/kg). Middle, Representative LA event indicated by a cyan arrow in the top LFP trace. Bottom, Representative “epileptic event” indicated by a red arrow in the top LFP trace. ***B***, LFP traces from mice injected with drugs. Detected LA events are labeled in cyan, and the entire epileptic events are labeled in red. ***C***, A raster plot of LA events (cyan) and epileptic events (red) in each drug-treated group. Each row represents each mouse, and each dot represents LA events or epileptic events (*n* = 4–5 mice in each group). For details of definition of LA events, see Figures 4-1 and 4-2.

We next monitored how hyperthermia affects neuronal activity in the postnatal mouse brain. To obtain cortical LFP signals from freely moving P11 mouse pups, we developed a recording system that minimized the weight of a recording device consisting of electrodes and an electrode interface to <0.3 *g* (Fig. 3*B*). LFP signals were recorded from the medial parietal association cortex or primary somatosensory trunk of mice (Fig. 3*C*,*D*) with video monitoring (Fig. 3*E*). Mice that were implanted with electrodes and treated with saline showed no significant difference in the latency to hyperthermia-induced clonic seizures (*n* = 4 mice, 1589 ± 234.2 s) compared to mice with no electrode implantation in the saline-treated group (*n* = 5 mice, 1619 ± 324.2 s; *t*_(8)_ = 0.071. *p* = 0.95, Student’s *t* test), confirming that our recording conditions do not affect seizure-related behavioral patterns. In Figure 3*F*, representative LFP traces before and during hyperthermia are presented. An LFP trace obtained from a mouse with chronic seizures demonstrates that epileptiform-like electrical discharges in the cortex were time-locked to behavioral seizures, consistent with a previous report (Dube et al., 2000). Artifacts and physiologic events were distinguished from epileptic events (Fig. 3*F*; for the detailed processes, see Materials and Methods and Fig. 4-1).

### GABA_A_ receptor modulators increase neuronal activity during FSs

In all recording sessions, LFP signals were recorded for 30 min to obtain baseline activity, and drugs were administered 15 min before hyperthermia induction (Fig. 3*A*). In the LFP signals, we first extracted LA events, which are considered to specifically represent neuronal discharges but not movement-related artifact signals (Fig. 4*A*; for a detailed validity of the threshold, see Materials and Method and Fig. 4-1). Based on our observation that the latency to chronic seizures was 337 ± 117.3 s and 207 ± 58.3 s in the pentobarbital- (37 mg/kg) and diazepam-treated (1 mg/kg) groups, respectively (Fig. 1*E*), we specifically focused on LFP patterns during the first 10 min period (Fig. 4). This is also because amplitudes of LA events increased especially in the early phase of hyperthermia period in GABA_A_ receptor modulators-treated groups (Fig. 4-2). Among the LA events (Fig. 4*A*, cyan traces), those in which the amplitude of the first peak was >3 mV were specifically extracted as epileptic events (Fig. 4*A*, red traces). Figure 4*B* shows representative LFP traces, including LA and epileptic events in individual drug-treated groups. All LA events, including epileptic events observed during this period, are summarized in a raster plot in Figure 4*C*. The results indicate that both diazepam and pentobarbital tend to increase the number of epileptic events, which are suppressed by bumetanide.

10.1523/ENEURO.0429-18.2019.f4-1 Figure 4-1. Detection of LA events. An LA event met the following three criteria: (i) The LFP envelope exceeded a threshold of the mean + 50×SD; (ii) The rise time was >10 and <200 ms; and (iii) The sharpness index exceeded 2. Among the LA events, events in which the amplitude of the first peak was >3 mV were specifically extracted as epileptic events. Figure 4-1, TIF file

10.1523/ENEURO.0429-18.2019.f4-2 Figure 4-2. Amplitudes of LA events. ***A***, A pseudo-colored raster plot of LA events in a pre-hyperthermia period. Each dot indicates a single LA event, and each row indicates each mouse in individual drug-treated groups. Each dot is color-coded according to the amplitude of each LA event. ***B***, The same as ***A***, but plotted for data obtained during hyperthermia. The black ticks indicate when the recording stopped. The initial 10 min period is magnified in time at the bottom. Figure 4-2. TIF file

### Bumetanide ameliorates GABA_A_ receptor modulator-induced epileptic events

**Figure 5.**
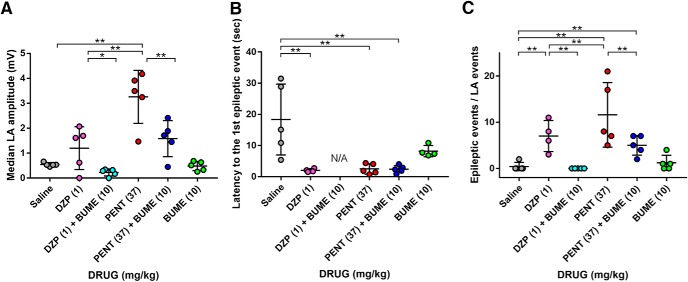
GABA_A_ receptor modulator-mediated epileptic events are suppressed by bumetanide. Across-group comparisons of the median amplitude of LA events (***A***), the latency to the first epileptic events (***B***), and the epileptic events/LA events (***C***) after the induction of hyperthermia. *p < 0.05, **p < 0.01, Tukey’s test; *n* = 4–5 mice.

First, we examined the effect of high-dose GABA_A_ modulators and bumetanide on LA amplitude. We found that median LA amplitude was increased in diazepam (not statistically significant) and pentobarbital groups compared with the saline group (Fig. 5*A*). We additionally confirmed that bumetanide alone did not affect the median LA amplitude, but it did significantly suppress the pentobarbital- and diazepam-induced increase in the amplitude (Fig. 5*A*).

Next, we examined the effects of high-dose GABA_A_ modulators and bumetanide on the latency and frequency of epileptic events. Consistent with the behavioral data that pentobarbital and diazepam shortened the latency to the first clonic seizures (Fig. 1*E*), the latency to the emergence of the first epileptic event was significantly shorter in the diazepam- and pentobarbital-treated groups, a phenomenon not blocked by bumetanide (Fig. 5*B*). We also found that bumetanide alone did not affect the number of epileptic events during 10 min but it completely blocked diazepam-induced epileptic events (Fig. 5*C*). These results indicate that the administration of high-dose GABA_A_ modulators enhance neuronal activity and susceptibility to seizures in mouse FSs at P11 and imply that the phenomena are in part mediated by depolarizing GABA signaling.

## Discussion

FSs are usually treated with GABA_A_ receptor modulators, but the effects of GABA_A_ receptor modulators on FSs are conflicting: most clinical studies reported that GABA_A_ receptor modulators are effective (Mamelle et al., 1984; Rosman et al., 1993; Knudsen, 1985; Guevara-González et al., 2018) but some studies reported the nonsignificant effects of GABA_A_ receptor modulators on FSs (Mckinlay and Newton, 1989; Uhari et al., 1995; Knudsen et al., 1996; Temkin, 2001). The question of whether GABA_A_ receptor modulators potentially activate immature neurons during early-life seizures remains unanswered. In the present study, we examined the question using a mouse model of FSs (Tao et al., 2016; Koyama, 2017) and found that high-dose GABA_A_ receptor modulators decrease the threshold to induce hyperthermia-mediated clonic seizures at P11 but not P14. These results suggest that GABA excitatory/inhibitory shift presumably takes place at ∼P11–P14 in mice.

We further determined that the deteriorating effects of high-dose GABA_A_ receptor modulators at P11 are likely mediated by enhanced excitatory GABA_A_ receptor signaling, because the GABA_A_ receptor modulator-mediated increase in the susceptibility to FSs was suppressed when the NKCC1 blocker bumetanide was coadministered (Fig. 1). These results suggest that prophylactic treatment with GABA_A_ receptor modulators could exacerbate seizure phenotypes in FSs probably via activating immature neurons when the GABA excitatory/inhibitory shift is not completed. However, it should be noted that the low-dose GABA_A_ receptor modulators suppressed seizures at P11 and that the sedation induced by high-dose GABA_A_ receptor modulators may exacerbate the seizure phenotype by preventing mouse pups from dissipating heat. Furthermore, as described previously, there are overwhelming evidences that GABA_A_ modulators are effective in human FSs and that our findings cannot be directly applied to human cases because the exact time point of GABA excitatory/inhibitory remain to be determined both in rodents and humans, making the direct comparison of the time point of GABA shift between rodents and humans difficult.

Though our results suggest that the GABA excitatory/inhibitory shift presumably takes place at ∼P11–P14 in a mouse model of FSs, it should be noted that the interspecies and inter-animal variability should be carefully considered. In the human, FSs occur between ∼1 month and ∼5 years of age (National Institutes of Health; International League Against Epilepsy; American Academy of Pediatrics). Comparing the development of the hippocampal formation between humans and rodents including neuronal formation, synaptic maturation, and afferent inputs, it was indicated that the first year of human life may be equivalent to P7–P14 in the rat (Avishai-Eliner et al., 2002, their Table 1). Baram et al. (1997) first developed a rat model of FSs using P10–P11 pups, and then the model was adapted to several strains of mice at P14–P15 (Dubé et al., 2005), based on the hippocampal development, threshold temperatures to induce FSs in normal children (Berg et al., 1992), and the reproducibility and reliability of stereotyped behavioral seizures. Thus, it is possible that the immaturity of hippocampal development in P11 mice contributes to the deteriorating effects of GABA_A_ receptor modulators in our study.

In the present study, we developed a novel LFP-video recording system to detect epileptic events in freely seizing neonatal mice. The electrode and electrode interface were minimized so that the interface does not interfere with mouse behaviors. During the induction of experimental FSs, we found LA events that were significantly eminent from the baseline LFP traces. A few previous studies performed LFP recordings during seizure events in postnatal mice (Chabrol et al., 2010; Dai et al., 2015), but the seizure-related behaviors and non-seizure-related behaviors have not been clearly distinguished. Finally, though most of previous studies have used rat pups for the study of electrophysiological properties of FSs probably because of the easily usable size of animals, our system uses mouse pups, which enables us to use a variety of transgenic animals.

It has been reported that FSs are accompanied by paroxysmal abnormality in LFP (Sofijanov et al., 1992; Kanemura et al., 2012). In our LFP recordings during hyperthermia, we observed high-voltage spikes with an average of 40 ms duration. Such spikes as well as stepwise sequential LFP changes have been also observed in human patients with FSs (Morimoto et al., 1991). Thus, the mouse FS model in our study likely mimics neuronal activities of human neonates during FSs. In humans, it is not realistic to precisely examine the correlations between LFP and behaviors during FSs because the onset of seizures is unpredictable and usually encountered at home. Thus, the LFP recoding system in mouse FS models is helpful to pharmacologically assess the mechanisms underlying FS-related neuronal activity and potentially the molecular backgrounds of FSs.

Using the recording system, we found that the GABA_A_ receptor modulators increased the neural circuit activity in the early phase of FSs, leading to the emergence of epileptic events (Fig. 5). It is likely that the GABA_A_ receptor modulators enhanced depolarizing GABA_A_ receptor signaling in the early phase of FS induction because coadministration of bumetanide suppressed neural circuit excitability. However, it should be noted that the effects of bumetanide on experimental FSs remain controversial (Koyama et al., 2012; Reid et al., 2013; Vargas et al., 2013; Ben-Ari et al., 2016; Hernan and Holmes, 2016). The brain levels of bumetanide after systemic administration are usually lower than the levels required for the effective activation of NKCC1 likely because bumetanide is highly ionized at physiologic pH, resulting in poor penetration into the brain. To increase the efficacy of NKCC1 activation, various prodrugs of bumetanide or alternative NKCC1 blockers with enhanced penetration into the brain have been developed and drugs that enhance Cl^−^ extrusion via KCC2 have also been investigated (Erker et al., 2016; Hampel et al., 2018). In the present study, we found that bumetanide alone did not affect the seizure phenotypes, but it suppressed the GABA_A_ receptor modulator-mediated deterioration of seizures in experimental FSs. It is known that benzodiazepines have lower toxicity than barbiturates generally (Fraser, 1998) and a previous work has reported that the coadministration of bumetanide and phenobarbital is effective for suppressing seizures in neonatal seizure models other than FS models (Dzhala et al., 2008; Cleary et al., 2013). Thus, our results suggest the potential for a combination therapy of bumetanide and diazepam for FSs treatment.

Our findings suggest that the use of high-dose GABA_A_ receptor modulators could exacerbate FS phenotypes likely via activating immature neurons when the GABA excitatory/inhibitory shift is not completed. It has been reported that GABA_A_ receptor modulators, especially barbiturates, could provoke neuronal damages that result in abnormal behaviors such as impaired cognition and depression in children (Brodie and Kwan, 2012; Kaushal et al., 2016). Thus, GABA_A_ receptor modulators such as benzodiazepines and barbiturates should be carefully used in children, and behavior should be monitored. It should be noted that our study focused on the induction phase of FSs in mouse pups and that the effects of GABA_A_ receptor modulator and bumetanide can be variable depending on the stage of FSs, the age, and the dose of reagents used. Finally, we showed that the coadministration of bumetanide with GABA_A_ receptor modulators could be effective for suppressing the induction and aggravation of FS phenotypes in mice. We hope that these findings can contribute to the establishment of therapeutic strategies to prevent the early-life FS-induced development of epileptic syndromes.

## Synthesis

Reviewing Editor: Tallie Z. Baram, University of California-Irvine Decisions are customarily a result of the Reviewing Editor and the peer reviewers coming together and discussing their recommendations until a consensus is reached. When revisions are invited, a fact-based synthesis statement explaining their decision and outlining what is needed to prepare a revision will be listed below. The following reviewer(s) agreed to reveal their identity: NONE. Note: If this manuscript was transferred from JNeurosci and a decision was made to accept the manuscript without peer review, a brief statement to this effect will instead be what is listed below.

**Synthesis of comments from the reviewers and from the editor that need attention and additional data from the authors**

Editor notes:

As indicated by the original reviewers of the J Neurosci submission, the study has merit.

However, the reviewers found several problems that are not fully addressed by the authors.

In addition, there is a discrepancy between this study and other studies from the authors' group that requires their attention: the age of the mice.

The authors have used P14 mice before (Tao, Neuroscience 2016), based on the literature (Dube 2005, Martin, 2010). The general assumption if that the state of hippocampal development of a P14 mouse is perhaps similar to that of a P10-P11 rat, as used by the authors in their terrific 2012 Nature Medicine paper.

Here the use P10 mice that are significantly less mature. Why? What are the results in P14 mice? It would be very important to know if the same results are found in older mice- or not. The authors are requested to provide these data.

Along the same lines, the message needs to be significantly clearer about the age of reversal of the chloride homeostasis in rodents and humans. There is evidence that neonatal seizures might not benefit from GABA-A modulators. However FS in humans occur later and the evidence is overwhelming that they do respond to GABA-A agonists. Thus, whereas the paper is important, the claims and the story need to be significantly toned down. We trust the clinical expertise of the authors will enable this. Note that credit should also be given to other reviews of FS in children- in addition to Dr Koyama's.

Point 2:

The authors need to seriously tone down their conclusions because they have not identified the mechanisms through which diazepam and barbiturates might exacerbate their FS. Specifically the doses of DZP and barbiturates used here are highly sedating. The sedation may be one of the reasons the pups cannot dissipate heat and have worse seizures. This notion also explains the opposing efefecs of low dose DZP (see comment 4 reviewer 2).

Reviewer #1

*Comment 2: The seizure activity in the DZP mice appears low despite the high mortality in this group*.

In the paper submitted to eNeuro, mortality is not indicated. This is critical because GABA modulators provoke lethargy, which prevents mice from handling the hyperthermia. Mortality for each group must be reported.

Please provide a table including mortality with all of the mice- NOT excluding post-hoc the low-dose diazepam, but including them as well.

*Comment 3: Similar datasets has been expanded to multiple figures. The authors should consider consolidating information from the same datasets. Many of the figures show the same results just presented differently*.

The extended data provided do not solve the Reviewer's comments. Please consolidate the data.

Reviewer #2

*General comments: In this ms the authors map the excitable features of hyperthermia induced febrile seizure using a novel method allowing for the recordings of eeg in young mice. The main premise of the study is to establish if the use of GABA-mediated inhibition should be contraindicated in FS. This is based on the idea that GABA can mediate excitatory potentials in early development due higher intracellular Cl-. They use a benzodiazepine and barbiturate to test this idea in mice. Both these drugs exacerbated the behavioural and electrophysiological FS phenotypes. This exacerbation could be reversed by using the NKCC1 blocker, bumentanide. Understanding the role of ‘excitatory GABA’ on seizure susceptibility is important and this data adds to a significant literature on the subject*.

Comment 1: Having a statement like “... alone are not always beneficial..” in the abstract is quite strong. Although the clinical motivation for the study is obvious, I don't think that the data as presented is sufficient to make these assertions. In fact the authors even make the point that they only look at the induction phase of FS...so the results need to be interpreted in that context. I would like to see the abstract softened -removing the inferred clinical conclusions. Also, the intro needs a little more added around the clinical evidence for using BZD (or not).

The authors' response is not sufficient to address the point.

Importantly there are MANY reasons why the sedating doses of diazepam and barbiturates worsen seizures in the authors' hands. See comment 4 below

*Comment 2: The authors look at P11 mice in their FS assay. What evidence do they have (other than the effect of Bumet) that GABA-mediated depolarisation occurs at this age? Most studies seem to suggest that by P11-12 that the switch has mostly happened. I think that the authors need to do some in vitro experiments to confirm this in their mice. On a similar note-it would be good to discuss when the switch might occur in humans and how this relates to when children have FS.*

The authors need to revise their answer (and please cite the Avishai-Eliner paper correctly)

The statement below must be removed- it is not based on facts:

“, it is plausible that the switching of GABA action is not completed in the brain when children have FSs.”

Avishai-Eliner S, Brunson KL, Sandman CA, Baram TZ (2002) Stressed-out, or in (utero)? Trends Neurosci. 25:518-524.

*Comment 4: I am trying to understand how 0.01mg/kg can completely protect from seizures while 1mg has no effect. The authors need to discuss this further*.

Answer 4: The interpretation of data was also difficult for us. In this revision, we have excluded the data of low dose treatment (DZP, 0.01 mg/kg and PENT, 0.37 mg/kg) to make the main points of this study clearer. Indeed, these low dose treatments were not very often used probably because it is not certain whether the concentration of drugs (especially when treated subcutaneously with low dose) in the postnatal brain are comparable between each mouse. It is also possible that the inverse results of DZP in low and high concentration may be due to degree of the enhanced excitatory GABAA receptor signalling. In the revised manuscript, we decided to present the data with higher dose (DZP, 1 mg/kg and PENT, 37 mg/kg) which have been more generally used in other studies.

**Excluding data is not an acceptable solution**. The authors are requested to re-inset the data and discuss it. Specifically low dose of DZP may augment GABA-A receptors without causing sedation. The sedation may be one of the reasons the pups cannot dissipate heat and have worse seizures.,
