# Supplementary figures and images for: The Pharmacological Assessment of GABAA Receptor Activation in Experimental Febrile Seizures in Mice
Source: eNeuro. 2019 Mar 4;6(1):ENEURO.0429-18.2019. doi: 10.1523/ENEURO.0429-18.2019 (PMC6498421; doi:10.1523/ENEURO.0429-18.2019)

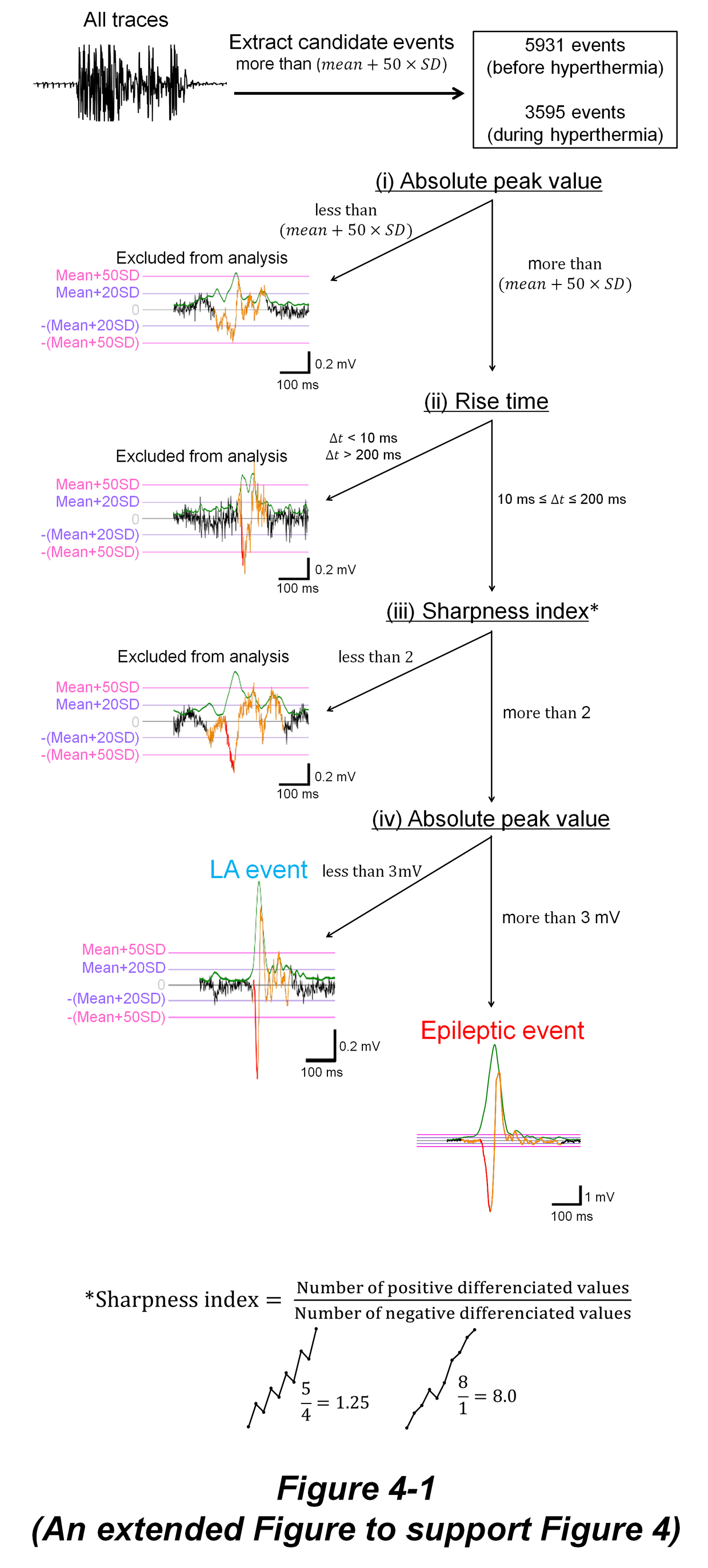

Supplement: Figure 4-1. — Detection of LA events. An LA event met the following three criteria: (i) The LFP envelope exceeded a threshold of the mean + 50×SD; (ii) The rise time was >10 and <200 ms; and (iii) The sharpness index exceeded 2. Among the LA events, events in which the amplitude of the first peak was >3 mV were specifically extracted as epileptic events. Figure 4-1, TIF file [file sup_enu-eN-TNWR-0429-18-s01.tif]

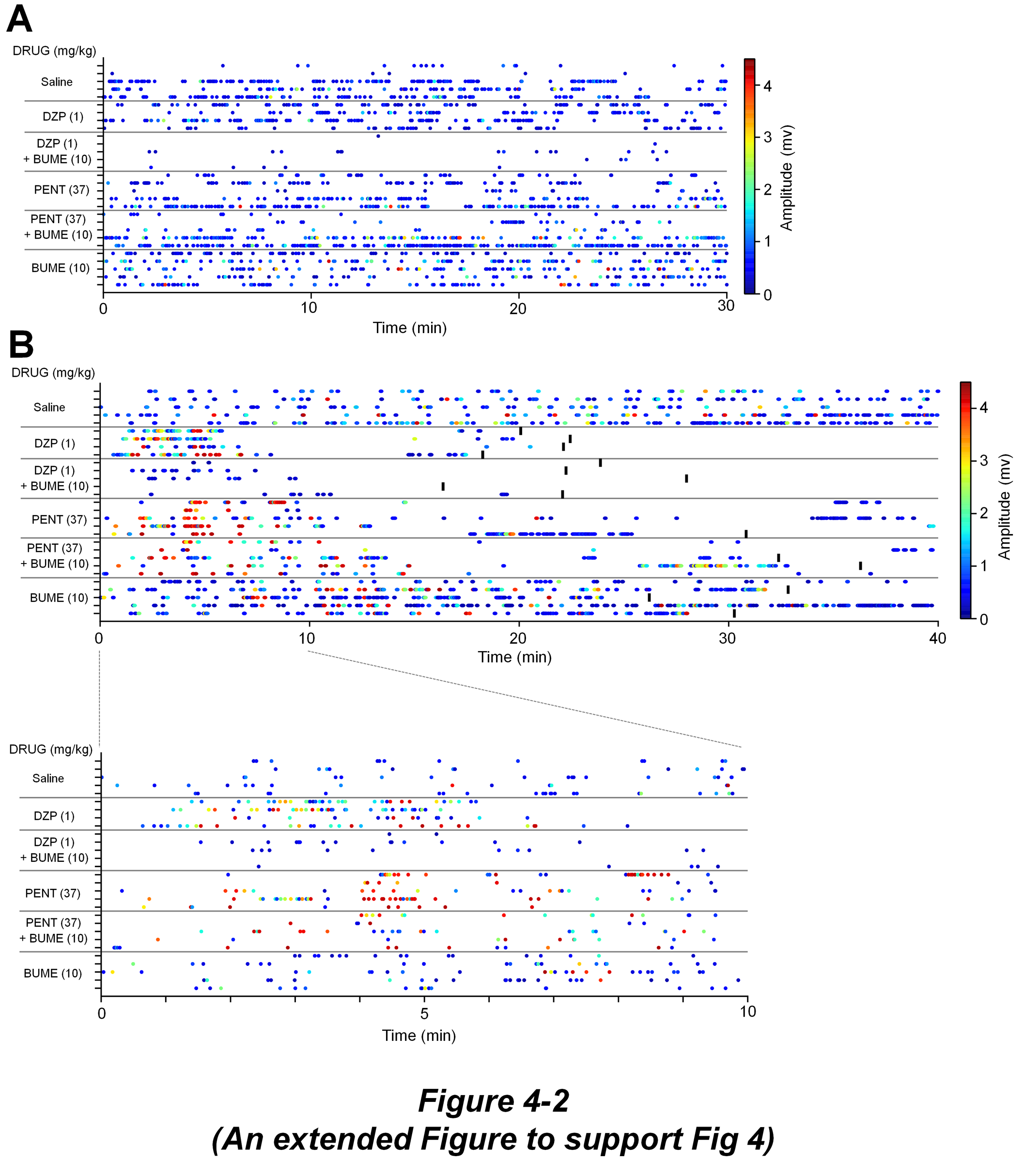

Supplement: Figure 4-2. — Amplitudes of LA events. A, A pseudo-colored raster plot of LA events in a pre-hyperthermia period. Each dot indicates a single LA event, and each row indicates each mouse in individual drug-treated groups. Each dot is color-coded according to the amplitude of each LA event. B, The same as A, but plotted for data obtained during hyperthermia. The black ticks indicate when the recording stopped. The initial 10 min period is magnified in time at the bottom. Figure 4-2. TIF file [file sup_enu-eN-TNWR-0429-18-s02.tif]
